# Assessment of safe handling practices among resident doctors in Jos University Teaching Hospital Plateau state, Nigeria

**DOI:** 10.4103/jomt.jomt_24_20

**Published:** 2021-04-28

**Authors:** Tolulope O. Afolaranmi, Zuwaira I. Hassan, Obadiah S. Pam, Lotanna M. Ugwu, Temidayo I. Oyegoke, Kayode K. Bello, Chundung A. Miner, Gabriel O. Ogbeyi

**Affiliations:** 1 Department of Community Medicine, University of Jos and Jos University Teaching Hospital, Jos Plateau State, Nigeria,; 2 Department of Community Medicine, Abubakar Tafawa Balewa University, Bauchi, Bauchi State, Nigeria,; 3 Faculty of Clinical Sciences, University of Jos, Jos Plateau State, Plateau State, Nigeria,; 4 Department of Community Medicine, Jos University Teaching Hospital Jos, Plateau State, Nigeria,; 5 Department of Epidemiology and Community Health, College of Health Sciences, Benue State University Makurdi, Nigeria

**Keywords:** Handling practice, resident doctors, Nigeria, safety precautions

## Abstract

**Background:**

Standard safety precautions are essential in health care delivery regardless of the presumed infectious state of the patients. Safe handling practices as detailed in the universal safety precaution guidelines are available to health care workers globally. However, there have been documentation of suboptimal adherence to it especially in the developing countries including Nigeria. Hence, this study was conducted to assess the level of safe handling practices and its determinants among resident doctors in Jos University Teaching Hospital, Plateau state, Nigeria.

**Methods:**

This was a cross sectional study conducted among 192 resident doctors using quantitative method of data collection in 2018.SPSS version 20 was used for data analysis with chi square test used to identify the determinants of safe handling practices. Crude odds ratio as well as 95% confidence interval were used with a p-value of < 0.05 considered statistically significant.

**Results:**

The mean age of the respondents in the study was 33 ± 3 years with 119 (62.0%) of the respondents being males. Good knowledge of infection, prevention and control was reported among 120 (62.5%) while 137 (71.3%) were found to have engaged in safe handling practices. Sex (OR = 4.5; 95% CI = 2.05–9.85) and level of knowledge (OR = 1.97; 95% CI = 1.05–3.72) were found as the determinants of safe handling practice.

**Conclusion:**

This study has brought to light the need for improvement in the level of compliance with safe handling practices as it is far from the optimum.

## INTRODUCTION

Hospital acquired infections are significant contributors to morbidity and mortality among health care workers globally.^[1]^Transmission of these infections occur when there is a breach in institutional or individual infection control practices.^[2]^ Healthcare workers are at higher risk of exposure to infectious agents either through direct contact with patients or from improper handling practices in the course of their work.^[3]^ Globally, about 3 million healthcare workers experience percutaneous exposure to blood borne pathogens each year and more than 90% of the infections from these exposures occurring in developing countries.^[2,3]^ Healthcare workers are at a greater risk of acquiring infections within the work place than others as estimated by World Health Organization that about 2.5% of HIV cases and 40% of hepatitis infections among health care workers are traceable of exposure at work.^[3]^

Standard safety precautions are essential in health care delivery regardless of the presumed infectious state of the patients, body fluids, secretions, work equipment and devices among others.^[1,3]^ The safe handling practices as detailed in the universal safety precaution guidelines are available to health care workers globally, however, there have been documentation of suboptimal adherence to it especially in the developing countries including Nigeria.^[4]^ Hence, this study was conducted to assess the level of safe handling practices and its determinants among resident doctors in Jos University Teaching Hospital, Plateau state, Nigeria.

## METHODS

### Study setting

This study was conducted in Jos University Teaching Hospital (JUTH), a tertiary health institution founded in 1975 and affiliated with the University of Jos.^[5,6]^ JUTH is a 600-bed capacity facility located in the Lamingo Area of Jos North Local Government Area (LGA).^[6]^ JUTH offers a vast variety of specialized services in the various aspects of healthcare, research and training and serves as a referral center to the surrounding states in the north central, parts of north western and north eastern part of Nigeria.^[6]^ JUTH being a tertiary health facility has following service delivery units; surgery, internal medicine, obstetrics and gynecology, pediatrics, community medicine, radiology, ophthalmology, pathology, laboratory medicine, otorhinolaryngology, anesthesia, psychiatry and dentistry among others.

### Study population

The study population comprised of all resident doctors undergoing specialty training in Jos University Teaching Hospital at the time of the study.

### Study design

A cross-sectional study design conducted between January and August 2018 to assess the level of safe handling practices and its determinants among resident doctors in Jos University Teaching Hospital, Plateau State Nigeria using quantitative method of data collection.

### Sample size estimation

The sample size for this study was determined using the appropriate sample size determination formula for a cross sectional study denoted below.^[7]^

$$n=\frac{Z^{2}\times Pq}{d^{2}}$$

Where *n* is the minimum sample size, *Z* is the standard normal deviate at 95% confidence interval (1.96), *q* is the complementary probability (1−*P*), *d* is the precision of the study set at 0.05 and p is the proportion of respondents with optimum compliance with safe handling practice from previous similar study being 86.96% (0.8696).^[8]^ This gave a minimum sample size of 192 after addition of 10% to cater for non, poor and or incomplete responses.

$$n=\frac{{\left(1.96\right)}^{2}\times 0.8696\times 0.1304}{{\left(0.05\right)}^{2}}=\frac{3.841\times 0.8696\times 0.1304}{0.0025}=174.4$$

### Criteria for inclusion in the study

All resident doctors in clinical departments for 1 year and upwards, who gave consent for participation were included in the study. Clinical departments were chosen in view of the fact that doctor–patient interaction was imminent and 1 year period was set as cut-off for inclusion as it would ensure that sufficient interaction with patient had occurred. Furthermore, clinical departments for the purpose of this study were those departments that provided both clinical services and in-patient care either in the teaching hospital complex or its outpost stations. These departments included psychiatry, surgery and its sub-specialties, internal medicine, obstetrics and gynecology, pediatrics, community medicine, ophthalmology, otorhinolaryngology, hematology, family medicine and dentistry.

### Sampling technique

A stratified sampling technique was used in view of the fact that the clinical departments had varied number of eligible resident doctors. A list of all the eligible resident doctors from the various departments was obtained, serialized and de-identified with unique departmental codes forming the sampling frame. Following which proportion to size technique was used to obtain the number of participants to be sampled from each of the departments. This was done by dividing the number of resident doctors who had met the inclusion criteria per department (psychiatry – 8, surgery and its sub-specialties – 44, internal medicine – 30, Obstetrics and gynaecology – 40, pediatrics – 20, community medicine – 41, ophthalmology – 5, otorhinolaryngology – 8, hematology – 7, family medicine – 32 and dentistry – 11) by the cumulative total number of all the eligible resident doctors in all the departments of interest (246) multiplied by the sample size of 192 for the study. This gave the following number of resident doctors to be sampled per department: psychiatry – 6, surgery and its sub-specialties – 34, internal medicine – 23, Obstetrics and gynaecology – 31, pediatrics – 17, community medicine – 32, ophthalmology – 4, otorhinolaryngology – 6, hematology – 5, family medicine – 25 and dentistry – 9. Thereafter, the respective departmental list was drawn and numbers were allocated to the all the eligible respondents in ascending order forming the departmental sampling frame from which computer generated table of random numbers was used to select determined number of resident doctors for each department respectively without replacement. They were then sampled in their respective departments daily while a departmental focal person helped in the retrieval of all the filled questionnaires for onward collection by the research assistants.

### Data collection

A semi-structured self-administer questionnaire adapted from a previous study comprising of three sections; socio-demographic characteristics, knowledge of infection, prevention and control and workplace handling practices.^[9]^ Four research assistants were trained on the content and method of administration of questionnaire prior to the commencement of the study by the principal researcher. The data collection instrument was pretested in among doctors in Plateau State Specialist Hospital Jos among 10% of the calculated minimum sample size in order to correct any ambiguity in the questionnaire as well as estimation of time and ease of administration of the questionnaires. Ethical clearance was obtained from Jos University Teaching Hospital Institutional Human Research Ethical Committee (JUTH/DCS/ADM/127/XXV/323). Written informed consent was obtained from all the respondents with confidentiality and anonymity of their responses assured and maintained.

### Scoring and grading of responses

A total of 6 stem questions were used to assess the level of knowledge with a maximum possible response of 12, out of which 6 were correct. Two (2) points were assigned to every correct response, while zero was assigned to incorrect response giving a maximum score of 12. A percentile graph of the score was plotted and scores less than the 50^th^ percentile was considered as poor knowledge while scores of 50^th^ percentile and above was considered as good knowledge.

Handling of work equipment (stethoscope) and outfit (ward coat, apron) was adjudged proper if respondents affirmed to dropping them in the health facility after daily work and not taking them home as well as ensuring they are properly laundered or cleaned.

Understanding of the concept of infection prevention and control was adjudged correct if the respondents mentioned information including the following; hand washing; use of barriers (such as gloves, gown, cap, mask), care with devices, equipment and clothing used during care; environmental control (such as surface processing protocols, health service waste handling); adequate discarding of sharp instruments including needles; and patients accommodation in accord to requirement levels as an infection transmission source.^[3,10]^

Handling practices was adjudged safe if the respondents provided favourable and affirmative responses of:

1. Always to question on use of gloves for contact with body fluids, non-intact skin and mucous membrane
2. Always to question on change gloves between patient contacts
3. Non recapping of needle and disposal in safety box to question on action taken on handling of sharps after giving injections or drawing blood from the patients
4. Always to question on change gloves between different procedures on the same patient
5. Never to question on reuse disposable gloves for procedures or examination
6. Always to use of ward coat, gown, apron during procedures likely to generate splashes of blood or body fluids
7. Always to use of face mask and eye protection for procedure likely to generate droplets/splash of blood or body fluid
8. Immediate cleaning and disinfection to question on splashing of blood, body fluid splash or stain on your personal protective equipments/work surfaces
9. Never to question on taking of any of the work outfit and equipment (ward coat, stethoscope etc.) to residential apartments after work
10. Always to question on hand washing with soap and running water after contact or procedure involving patients.

### Data analysis

The data obtained were processed and analyzed using SPSS version 20 where qualitative socio-demographic characteristics of the respondents such as age group, sex, duration of practice (years) as well as measures of levels of knowledge of infection, prevention and control and workplace handling practices were expressed in frequency and percentage. Mean ± standard deviation was used as summary indices for age of the respondents and knowledge score while median and Interquartile Range (IQR) used for duration of practice in view of the fact that it was skewed. Chi-square (*χ*^2^) statistical test was used to determine the association between explanatory variables such as age, sex, duration of practice, prior attendance of training on infection prevention and control and level of knowledge etc. and handling practice. Crude odds ratios was used as point estimate with 95% confidence interval used as the interval estimate while a probability value of less than 0.05 was considered statistically significant.

## RESULTS

The mean age of the respondents in this study was 33±3 years with 51 (26.6%) of them being 36 years and above. With regards to the sex of the respondents, 119 (62.0%) were males and 103 (53.6%) married. Assessment of the duration of practice of medicine revealed that 123 (64.1%) had been practicing for 5 years or less with a median duration of 4 (IQR: 3–7) years [Table 1].

In the assessment of awareness of nosocomial infections among the respondents, 158 (82.3%) mentioned HIV/AIDS while viral hepatitis infections, pulmonary tuberculosis and viral haemorrhagic diseases were mentioned by 147 (76.6%), 83 (43.3%) and 136 (70.8%) respectively. Furthermore, only 38% of the respondents demonstrated good understanding of the concept of infection prevention and control as 86 (44.8%), 63(32.8) and 97 (50.5%) affirmed that hand washing before and after handling patients, proper handling and disposal of sharps and use of appropriate barrier methods are the elements of infection prevention and control. Overall, good level of knowledge of infection prevention and control was demonstrated by 120 (62.5%) of the study participants as shown in Table 1.

Assessment of handling practices of the respondents in the course of discharging medical services revealed that 80 (41.6%) of the participants would immediately clean or remove their work outfits if blood, blood products or body fluids splash on them in the course of performing non-surgical procedures. Additionally, 30 (15.7%) would remove such work outfits at the close of work and 29 (15.1%) being indifferent about taking any specific action. Furthermore, 70 (36.5%) of the participants affirmed to leaving their non-disposable work outfit in their respective call rooms at the close of work while 47 (24.5%) would keep them in the car and 26 (13.5%) would take them home. With regards to the use of hand gloves, 170 (88.5%) always used gloves when handling patients regardless of the disease conditions while 122 (63.5%) always used face mask for clinical procedure suspected to be infectious. Additionally, safe handling practice was reported in 137 (71.3%) while the corresponding 55 (28.7%) adjudged to be engaged in unsafe work handling practices [Table 1].

Safe work handling practice was found to be significantly influenced by sex of the participant with its odds among the females being about 4.5 times (95% Cl: 2.05–9.82) that of their male counterparts. Also, the odds of safe work handling practice among those with good knowledge of infection prevention and control was about 2 times (95% Cl: 1.05–3.72) those adjudged to have poor knowledge [Table 2].

## DISCUSSION

Standard safety precautions represent the minimum infection prevention practices employed by health care workers to patient care in any health care service delivery setting regardless of infection status of the patients. These practices are established to protect the health care workers from acquiring infections from their patients or transmitting the infections to their patients and other health care workers. These precautionary measures include but not limited to hand hygiene, use of personal protective equipment such as gloves, gowns and masks, needle safety, safe handling of potentially contaminated equipment or surfaces and proper disposal of sharps, body fluids and other clinical wastes.^[10]^ Imperatively, the adequacy of the knowledge of these standard safety precautions by health care workers is essential to achieving a safe work environment devoid of transmission of nosocomial infections. In this study, more than half of the participants demonstrated good knowledge of infection prevention and control as entrenched in the standard safety precaution which is in synergy with findings of studies conducted in Nigeria and Jamaica.^[1,8,10–14]^ This similarity could be attributable to the fact that health care institutions globally have demonstrated renewed commitment to ensuring that health care workers are equipped with the necessary information on infection prevention and control through structured training, supportive supervision and scholar exchange in the face of global infection threats and emerging infections. Furthermore, improved access to continued medical education on standard safety precautions could also be a factor. Importantly, this study and others as cited have brought to light significant gaps in knowledge of standard safety precaution as no study reported good knowledge in all the participants indicating that efforts have to be put in place to ensure that this knowledge gap is filled using context based methods and strategies.

In furtherance, other studies have also reported varied levels of knowledge of infection, prevention and control lower than that of this study with some reporting abysmally low levels. ^[12,15–21]^ This variation could be due to the fact that, the study participants cut across all cadres of health care workers in some studies as against our study where the participants were only medical doctors in residency training. More so, this study was conducted in a tertiary health institution while some of the other studies were carried out at a lower level of health care service delivery. Additionally, variation in the heath institutional training structure as well as individual’s disposition to knowledge acquisition and self-improvement might be contributory. Therefore, the implication of this varied level of knowledge of infection prevention and control among health care workers might mean that some health facilities could be still be serving as hubs of infection transmission if some of the health care workers do not have the sufficient knowledge base for strict compliance with standard safe handling practices.

Safe handling practices in compliance with the standard precautions by health care workers are essential in the prevention of acquisition and transmission of nosocomial infections.^[8,22]^In this study, most of the participants were adjudged to have engaged in safe handling practices while providing health care services with shared similarities with findings of other Nigerian studies.^[10,11,14]^However, findings of studies carried out in Uganda and Nigeria revealed a higher level of compliance with safe handling requirements while lower levels were reported in some other studies.^[8,16,17,18,23,24]^ The reported level of safe handling practices in our study could be due to that fact it was conducted in a tertiary health institution where there are established structures built into the systems to ensure and promote standard and safe handling practices no matter how elementary in addition to the fact that hierarchy of supervision also exists. Furthermore, the role of environmental, structural and personal/individual factors as regards the engagement in safe handling practices across various study settings needs to be further explored. Additionally, self-reported approach to assessment of handling practices was employed in this study as used in some of the other cited studies which may not give the true reflection of the actual situation, It is therefore imperative for further studies employing observation approach to the assessment of handling practices be carried out while ensuring that Hawthorne effect is mitigated against to the barest minimum.

Sex and knowledge of infection prevention and control were identified as the determinants of safe handling practices in this study which is further corroborated by findings of other studies which identified same factors in addition a variety of other factors such as age, years of experience as health care workers, positive attitudes, professional cadre, type of heath facility, prior training and availability of personal protective wares.^[10,11,13,14,17,19]^ The diversity of the identified factors influencing safe handling practices during health care service provision further reiterates the fact that constellation of factors needs to be considered when structuring intervention targeted at addressing poor handling practices. More so, this study was limited in its ability to conduct facility audit of availability and adequacy of structures and materials needed for enhancement of safe handling practices, hence paving ways for further studies in this regard.

In conclusion, this study has brought to light the need for improvement in the level of compliance with safe handling practices as it is far from the optimum while sex and knowledge of infection prevention and control can be used the fulcrum in structuring intervention for improving it among other measures.

## Figures and Tables

**Table 1:** Characteristics, knowledge and practice of safe handling among the respondents

Variables	Frequency (*n* = 192)	Percentage
AGE (Years) > 35	141	73.4
Males	119	62.0
Married	103	53.6
Duration of practice > 5 years	123	64.1
Attended training on infection prevention and control within the last 5 years	38	19.8
Awareness of common infections transmitted in the hospital*		
HIV/AIDS	158	81.9
Hepatitis viral infections	147	76.2
Pulmonary tuberculosis	83	43.0
Viral haemorrhagic Disease	136	70.5
Others**	36	18.7
Good understanding of the concept of infection prevention and control	73	38.0
Elements of infection, prevention and control mentioned *		
Hand washing before and after handling patients	86	44.8
Proper handling and disposal of sharps	63	32.8
Use of appropriate barriers	97	50.5
Proper handling of work equipment and clothing	31	16.1
Others***	60	31.3
Good level of knowledge	120	62.5
Actions taken when blood, blood products and body fluid splash on work outfit and equipment		
Immediate cleaning/removal	80	41.6
Removal after completion of procedure	53	27.6
Removal at close of work	30	15.7
Places for keeping of continuous use work outfit		
Call room	70	36.5
Car	47	24.5
Home	26	13.5
Others^+^	49	25.5
Handling of non autodestruct syringe and needle after use		
Dispose without recapping	80	41.7
Removal of needles from syringes and dispose	81	42.2
Bend the needles and dispose	13	6.8
Recapping of needles and dispose	18	9.3
Use of gloves when handling patients		
Always	170	88.5
Often	20	10.5
Seldom	2	1.0
Never	0	0.0
Wearing of work outfit for procedures and clinical activities		
Always	161	83.9
Often	29	15.1
Seldom	2	1.0
Never	0	0.0
Using of face mask for clinical procedures apart from surgeries		
Always	122	63.5
Often	35	18.2
Seldom	31	16.1
Never	4	2.1
Safe level of handling practice	137	71.3

* Multiple responses elicited,

** Diseases with exudative skin lesions,

+ Outfit bags, Laundry basket, Hospital locker,

*** patients admitted in accordance to the safety requirement levels, use of proper surface cleaning protocol and disinfection, proper hospital waste management

**Table 2:** Relationship between characteristics of the respondents and handling practice

	Handling practice					
	Safe	Not Safe				
Characteristics	Freq (%)	Freq (%)	Total	*χ*^2^	*P*-value	COR (95Conf. Interval)
Age (year)						
≤35	102 (72.3)	39(27.7)	141	0.253	0.615	1.20 (0.60–2.39)
>35	35 (68.6)	16 (31.4)	51			
Sex						
Female	64 (87.7)	9 (12.3)	73	15.342	<0.001	4.48 (2.05–9.82)
Male	73 (61.3)	46 (38.7)	119			
Marital status						
Married	76 (73.8)	27 (26.2)	103	0.643	0.423	1.29 (0.69–2.41)
Single	61 (68.5)	28 (31.5)	89			
Duration of Practice (years)						
≤5	91 (74.0)	32 (26.0)	123	1.152	0.282	1.42 (0.75–2.69)
6 and above	46 (66.7)	23 (33.3)	69			
Attendance of training						
Attended	24 (63.2)	14 (36.8)	38	1.557	0.212	0.62 (0.30–1.31)
Not attended	113 (73.4)	41 (26.6)	154			
Level of knowledge						
Good	92 (76.7)	28 (23.3)	120	4.418	0.036	1.97 (1.05–3.72)
Poor	45 (62.5)	27 (37.5)	72			

COR=Crude Odds Ratio, C.I= Confidence interval

## References

[1] Vaz K, McGrowder D, Alexander-Lindo R, Gordon L, Brown P, Irving R. Knowledge, awareness and compliance with universal precautions among health care workers at the University Hospital of the West Indies, Jamaica. Int J Occup Environ Med 2010;1:171–81. 23022806

[2] World Health Organization (WHO). Practical guidelines for infection control in healthcare facilities. Publication of Western Pacific Regional office Manila and South East Asia Regional office New Delhi. Available from: https://apps.who.int/iris/handle/10665/206946. [Cited 2020 January, 20].

[3] Buowari OY. Universal Precautions: a review. The Nigerian Health Journal 2012;12:68–73.

[4] Moyo GM. Factors influencing compliance with infection prevention standard precautions among Nurses working at Mbugathi district hospital, Nairobi, Kenya. A dissertation submitted in partial fulfillment for the requirement of the award of degree of Master of Science in Nursing (Medical Surgical Nursing) of the University Nairobi. 2013. Available from: http://erepository.uonbi.ac.ke/bitstream/handle. [Cited 2019 November, 20].

[5] National Bureau of Statistics. 2006 Population Census. Available from: http://www.nigerianstat.gov.ng. [Cited 2019 September, 27].

[6] Jos University Teaching Hospital. About JUTH. Available from: http://www.juth.org.ng. [Cited 2019 December, 23].

[7] Ibrahim T Sample size determination. In: Research methodology and dissertation writing for health and allied health professionals. 1st edition Abuja, Nigeria: Cress Global Link Limited. 2009;75–9.

[8] Frank MD, Robinson-Bassey GC, Ibudeh SE. Knowledge and compliance with standard precautionary measures among nurses in Madonna University Teaching Hospital, Elele, Rivers State. International Journal of Nursing Didactics 2016;6:21–26. 10.15520/ijnd.2016.vol6.iss3.144.21-26.

[9] Agu PU, Ogboi SJ, Ezegwu EC, Okeke TC, Aniebue PN. The knowledge, attitude and practice of universal precaution among rural primary health care workers in Enugu South-East, Nigeria. World Journal of Pharmacy and Pharmaceutical Sciences 2015;4:109–25.

[10] Adebimpe WO. Knowledge, attitude, and practice of use of safety precautions among health care workers in a Nigerian tertiary hospital, 1 year after the Ebola Virus Disease epidemic. Ann Glob Health 2016;82:897–902. doi:10.1016/j.aogh.2016.07.004. 28283144

[11] Johnson OE, Assi AE, Bakpo IJ, Harrison UE, Angba FE, Okon MA. Knowledge and practice of standard precautions among health care workers in a secondary facility in southern Nigeria. International Journal of Biomedical Research 2019; 10:e5033. doi: 10.7439/ijbr.v10i1.5033.

[12] Abdulraheem IS, Amodu MO, Saka MJ, Bolarinwa OA, Uthman MMB. Knowledge, awareness and compliance with standard precautions among health workers in north eastern Nigeria. Journal of Community Medicine and Health Education 2012;2:131. doi:10.4172/jcmhe.1000131.

[13] Arinze-Onyia SU, Ndu AC, Aguwa EN, Modebe I, Nwamoh UN. Knowledge and practice of standard precautions by healthcare workers in a tertiary health institution in Enugu, Nigeria. Niger J Clin Pract 2018;21:149–55. doi:10.4103/njcp.njcp_69_17 29465047

[14] Afolaranmi TO, Hassan ZI, Bello DA, Oyebode T, Chingle MP. Assessment of knowledge and practice of standard safety precautions among Primary Health Care Workers in Plateau State North Central Nigeria. International Journal of Biomedical Research 2017;8:187–93. 10.7439/ijbr.

[15] Chaudhuri S, Baidya OP, Singh TG. Universal precaution: practice among doctors in a tertiary care hospital in Manipur. International Journal of Research in Medical Sciences 2016;4:606–9. 10.18203/2320-6012.ijrms20160324.

[16] Fayaz SH, Higuchi M, Hirosawa T, Sarker MA, Djabbarova Z, Hamajima N. Knowledge and practice of universal precautions among health care workers in four national hospitals in Kabul, Afghanistan. J Infect Dev Ctries 2014;8:535–42. Published 2014 Apr 15. doi:10.3855/jidc.4143 24727521

[17] Esu I, Okeke C, Gobir A. Factors affecting compliance with standard precautions among healthcare workers in public hospitals Abuja, Nigeria. International Journal of Tropical Diseases & Health 2019;36:1–11. doi: 10.9734/IJTDH/2019/v36i230141

[18] Jain M, Dogra V, Mishra B, Thakur A, Loomba PS. Infection control practices among doctors and nurses in a tertiary care hospital. Annals Tropical Medicine and Public Health 2012;5:29–33.

[19] Tobin EA, Asogun DA, Odia I, Ehidiamhen G. Knowledge and practice of infection control among health workers in a tertiary hospital in Edo state, Nigeria. Direct Research Journal of Health and Pharmacology 2013;1:20–27.

[20] Bamigboye AP, Adesanya AT. Knowledge and practice of universal precautions among qualifying medical and nursing students: a case of Obafemi Awolowo University Teaching Hospitals complex, Ile-Ife. Research Journal of Medicine and Medical Sciences 2006;1:112–6.

[21] Okechukwu EF, Modteshi C. Knowledge and practice of standard precautions in public health facilities in Abuja, Nigeria. International Journal of Infection Control 2012; v8:i3 doi: 10.3396/ijic.v8i3.022.12

[22] Garner JS. Guideline for isolation precautions in hospitals. The Hospital Infection Control Practices Advisory Committee. Infect Control Hosp Epidemiol 1996;17(1):53–80. doi:10.1086/647190 8789689

[23] Mpamize G Adherence to universal precautions in infection prevention among health workers in Kabarole district. Journal of Health, Medicine and Nursing 2016;26:144–55.

[24] Osuala EO, Oluwatosin OA. Infection control by nurses in selected hospitals in Anambra State, Nigeria. Tropical Journal of Medical Research 2017;20:53–60. Doi: 10.4103/1119-0388.198122

